# Universal Molecular Triggers of Stress Responses in Cyanobacterium *Synechocystis*

**DOI:** 10.3390/life9030067

**Published:** 2019-08-20

**Authors:** Kirill S. Mironov, Maria A. Sinetova, Maria Shumskaya, Dmitry A. Los

**Affiliations:** 1 Department of Molecular Biosystems, K.A. Timiryazev Institute of Plant Physiology, Russian Academy of Sciences, Botanicheskaya street 35, 127276 Moscow, Russian; 2 Department of Biology, School of Natural Sciences, Kean University, 1000 Morris Ave, Union, NJ 07083, USA

**Keywords:** cyanobacteria, membrane fluidity, ROS, stress responses, universal triggers

## Abstract

Systemic analysis of stress-induced transcription in the cyanobacterium *Synechocystis* sp. strain PCC 6803 identifies a number of genes as being induced in response to most abiotic stressors (heat, osmotic, saline, acid stress, strong light, and ultraviolet radiation). Genes for heat-shock proteins (HSPs) are activated by all these stresses and form a group that universally responds to all environmental changes. The functions of universal triggers of stress responses in cyanobacteria can be performed by reactive oxygen species (ROS), in particular H_2_O_2_, as well as changes in the redox potential of the components of the photosynthetic electron transport chain. The double mutant of *Synechocystis* sp. PCC 6803 (*katG/tpx*, or *sll1987/sll0755*), which is defective in antioxidant enzymes catalase (KatG) and thioredoxin peroxidase (Tpx), cannot grow in the presence of exogenous hydrogen peroxide (H_2_O_2_); and it is extremely sensitive to low concentrations of H_2_O_2_, especially under conditions of cold stress. Experiments on this mutant demonstrate that H_2_O_2_ is involved in regulation of gene expression that responds to a decrease in ambient temperature, and affects both the perception and the signal transduction of cold stress. In addition, they suggest that formation of ROS largely depends on the physical state of the membranes such as fluidity or viscosity. In cyanobacteria, an increase in membrane turnover leads to a decrease in the formation of ROS and an increase in resistance to cold stress. Therefore: (1) H_2_O_2_ is the universal trigger of stress responses in cyanobacterial cells; (2) ROS formation (in particular, H_2_O_2_) depends on the physical properties of both cytoplasmic and thylakoid membranes; (3) The destructive effect of H_2_O_2_ is reduced by increasing of fluidity of biological membranes.

## 1. Introduction

Cyanobacteria are oxygenic phototrophic prokaryotes that represent the most ancient group of organisms able to perform oxygenic photosynthesis. Cyanobacterial survival and successful function under stress both depend on their capability to adapt to environmental changes. Recent progress made in cyanobacterial molecular signaling research has led to our improved understanding of both basic mechanisms of perception and transduction of stress signals as well as evolution of certain regulatory systems. Such knowledge is useful for related fields, e.g., antimicrobial drug design or stress research in food and industrial crops. With the growing interest in biofuel production in cyanobacteria, which often involves modification of both genetic structure and growth conditions of natural cyanobacterial strains, the response and adaptation of these organisms to multiple internal and external stress factors is of a special interest.

Earlier studies [1] show that cyanobacterial cellular response to various types of abiotic stress (heat shock, oxidative stress, salt stress, hyperosmotic stress, high-intensity illumination, ultraviolet (UV) irradiation) is universal, and includes a transient decrease of cytoplasm volume [2], a decrease in photosynthetic activity [3], and an increase in respiration [4]. In addition, reverse genetics and transcriptome analysis performed with DNA microarray and RNA-seq technologies, suggest that different types of stress might not only cause similar cellular changes, but also induce expression of the same sets of genes in cyanobacteria [1], or activate multisensory proteins that can perceive abiotic stresses regardless of their nature [5,6]. For example, inactivation of a sensory histidine kinase, Hik33, alters cell responses to all cold, salt, hyperosmotic, and oxidative stresses in the cyanobacterium *Synechocystis* sp. PCC 6803 (hereafter referred to as *Synechocystis*) [7]. Another histidine kinase, Hik31, is involved in regulation of autotrophic growth [8], responses to light [9,10], and resistance to divalent cations (Cd^2+^, Cu^2+^, and Zn^2+^) [11,12]. Histidine kinase Hik34 acts as a negative transcriptional regulator during heat shock [13], a positive regulator under salt or hyperosmotic stress, and an autoregulator under oxidative stress [14].

These observations suggest that different stressors may generate some universal intracellular signal(s) that triggers adaptive cell responses. Here we review the attempts to identify those signals by systematic analysis of cyanobacterial stress transcriptomes, and the efforts to evaluate the hypothesis of universal signals that trigger stress responses in cyanobacteria.

Unfortunately, there is a limited set of genome-wide data on other cyanobacterial strains subjected to abiotic stressors. Transcriptomic analyses are limited to some *Anabaena* and *Synechococcus* strains subjected to ion or nitrogen limitations. Such nutrient limitation data are incomparable to the data on temperature, light, salt or osmotic stress. Therefore, here we focus on the model strain *Synechocystis* keeping in mind that the discovered patterns can be attributed to other cyanobacterial and bacterial species.

## 2. Systemic Analysis of Cyanobacterial Stress Transcriptomes

*Synechocystis* is a model organism used in cyanobacterial research, especially in the stress response field. In general, optimal growth of *Synechocystis* occurs at temperatures of 30–35 °C, under continuous illumination of 70–120 μmol quanta m^−2^·s^−1^, and final CO_2_ concentration in bubbling air of 1%–1.5%. Various shifts from the optimal conditions result in stress exposure, under which the gene expression is widely researched.

### 2.1. Genes Induced by Heat Stress

In *Synechocystis*, heat stress is caused by exposure to 42–45 °C. *Synechocystis* cells are able to survive a 24-h incubation at 44 °C, pointing to their high thermal resistance [4].

Heat stress has been shown to transiently induce approximately 80 genes with the induction factor (IF) ≥ 2 within the first 15–30 min [13,15,16] (Table 1). When a cell culture is heat stressed for a longer period, in most cases, no increase in transcription is detectable. Many genes are activated just within few minutes from the beginning of the stress treatment, and then, within 30–60 min, their transcription declines and returns to the baseline level recorded before stress exposure [13,15].

The list of genes for the heat shock proteins (HSPs) includes *hspA*, *groES*, *groEL1*, *groEL2*, *dnaJ*, *htpG*, *dnaK2*, and *clpB1* (chaperones). These genes demonstrate the highest transcriptional upregulation, together with *htrA* (protease), *sigB* and *sigD* (RNA polymerase σ factors), *hik34* (heat-sensing histidine kinase), *sodB* (superoxide dismutase), and several others. Some of the products of these genes tightly interact with each other, for example, RNA polymerase σ factor B (SigB) is necessary for the normal synthesis of the *hspA* mRNA and cell survival upon a short-term heat [17,18] or salt [19] stress, and the activity of SigB is controlled by Hik34 [13]. SigD regulates the expression of several genes essential for adaptation to oxidative stress induced by high NaCl concentrations [20], and its activity is controlled by a pair of histidine kinase Hik33 and response regulator Rre31 [19].

Apart from the high temperature, the transcription of HSP genes is induced by hyperosmotic [21] or salt stress [19,22,23], strong light [24], UV-B [25], oxidative stress [14,26], and low pH [27,28]. Only 7 out of 80 genes demonstrate a specific response to heat shock (Table 1, labeled with asterisk). The function of more than a half of genes induced by heat shock is still unknown. Therefore, one cannot assume that HSP genes are specific heat shock responders; since their expression changes after a variety of stress factors, HSPs (and their genes, respectively) are rather considered “general stress proteins” (GSPs) [5,6]. Hence, hereafter we will refer to known HSPs as GSPs.

### 2.2. Genes Induced by Strong Light and Ultraviolet-B (UV-B)

Similarly to heat shock, strong light (here, a shift from optimal intensity of 20 to 400 μmol photons/m^2^ s) causes a rapid (within 15–20 min) but transient increase in genes transcription. More than 100 genes are induced with IF ≥ 2, including GSP genes: *hspA*, *clpB1*, *dnaK2*, *htpG*, *groES*, *groEL1*, and *groEL2*; *hli* (high light inducible) family; genes for NADPH^+^ dehydrogenase subunit (*ndh*); and genes for the components of CO_2_-concentrating mechanism (*ccm*), and ribulose-1,5-bisphosphate carboxylase (RuBisCO) [24,29]. Transcription of several genes, such as *groESL* and *groEL2*, remains at high level even after several hours of exposure to strong light.

Strong light stimulates production of reactive oxygen species (ROS) which are expected to upregulate expression of genes involved in neutralizing ROS. However, out of several ROS-neutralizing genes only superoxide dismutase gene (*sodB*) and, to a certain extent, glutathione peroxidase gene (*gpx1*) are induced by strong light. In contrast, transcription of catalase (*katG*) and thioredoxin peroxidase (*tpx*) genes have not been found to change during strong light stress [23,26].

UV-B upregulates nearly 50 genes with IF ≥ 2 [24] including genes for GSPs, a precursor of the D1 protein of PS II (*psbA2* и *psbA3*), proteases involved in D1 maturation and degradation (*ctpA* и *ftsH*), *nblB* for a phycobilisome disassembly protein [28], *hik34*, *sigB*, and *sigD* (Table 1).

### 2.3. Genes Induced by Salt and Hyperosmotic Stress

Salt stress (0.5 M NaCl for 15-20 min) induces more than 100 genes in *Synechocystis* with IF > 4 [19,22], including genes for GSPs, such as HtrA protease (*htrA*), *sigB* and *sigD*, and superoxide dismutase (*sodB*). Specifically, NaCl induces genes for ABC-type transporters (*cbiQ*, *norM*, and *ycf85*), quinone synthesis (*menB*, *menG*, and *ubiH*), biotin (*birA*) and riboflavin (*ribF*) metabolism, NAD biosynthesis (*nadC*), protein acetylation (*rimI*), protease HhoB, translation initiation factor (*infC*), and some genes with unknown functions [5,22,23].

Hyperosmotic stress (0.5 M sorbitol for 15–20 min) induces a similar set of non-specific stress-response genes (GSPs, *sig*, etc.) [21]. Such a uniform stress response is explained by a decrease in a cell volume due to rapid water outflow from the cytoplasm during the initial stage of both stresses [30,31]. Only few genes respond specifically to the sorbitol treatment; these include genes for phosphate (*pstS*) and nitrate/nitrite (*ntrABC*) transporters, lipid metabolism (*sll0208* and *sll1377*), periplasmic protease (*hhoA*) and several genes with unknown functions [5,21,32].

### 2.4. Genes Induced by Cold Shock and Other Stressors

**Figure 1:**
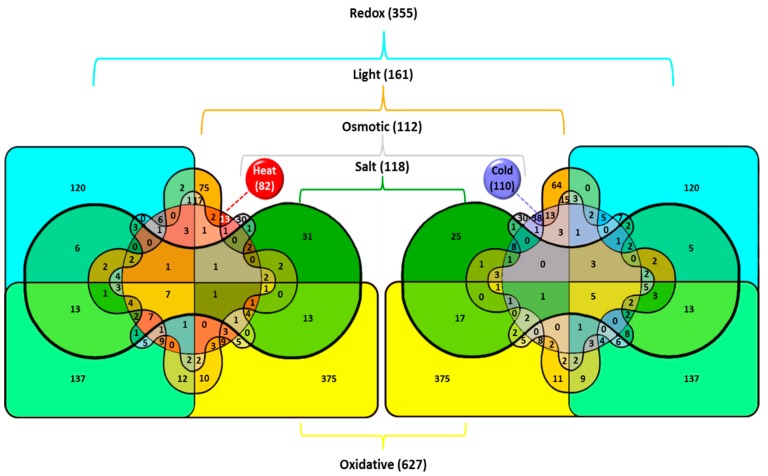
Venn diagrams illustrating the effects of various stresses on the induction of gene transcription in the cyanobacterium *Synechocystis*. Total number of genes upregulated in particular stress is shown in parentheses. The number of genes induced by the particular stress or by several stress types is shown without parentheses. Venn diagrams were generated with InteractiVenn at http://www.interactivenn.net. Reproduced from [5] with permission.

At the early stages of temperature stress, the genes whose transcription is induced by cold are different from those induced by heat (Figure 1). Expression of about 100 genes (Table 2) increases more than twice after lowering the ambient temperature to 22 °C for 30 min [15,33]. These genes belong to several major operational groups, as summarized in [34]:

(1) signal perception and transduction;

regulatory genes for cold stress are limited to two histidine kinases (light-regulated Hik3 [35] and multifunctional Hik31 [8,9,10,11,12]), two genes for the DNA-binding transcriptional regulators (*rre5* and *sfsA*) [36], and a gene for the RNA polymerase σ-factor *sigD*. Hik31 is activated by Cu^2+^, released as a result of oxidation of plastocyanin by photosynthetically producing electrons, and triggers the connected regulatory cascade [11,12]. Rre5 is a PatA-subfamily response regulator, which is involved in CO_2_ uptake and is associated pH homeostasis [34]. Transcriptional factor SfsA regulates sugar catabolism [37], and SigD is involved in light-dependent redox regulation of transcription [18,38].

(2) transcription and translation;

genes from this group encode proteins that prevent issues with transcription and translation caused by cold. For example, a hindrance such as antisense transcription is reduced with the assistance of NusG (a transcriptional terminator) [39], ribosome protein misfolding is prevented by Tig (a chaperone trigger factor), and stalled ribosomes are rescued with the assistance of SmpB [40]. Interestingly, an RNA helicase CrhR from this group participates in redox regulation of the plastoquinone (PQ) pool [41,42].

(3) photosynthesis and respiration—these genes will be discussed further;

(4) cell wall and membrane maintenance;

three of four genes for the fatty acid desaturases (FADs) induced by cold treatment are the key enzymes modulating cold-dependent changes in membrane fluidity. In cyanobacteria, enzymatic dehydrogenation occurs in fatty acids (FAs) of glycerolipids. Lowering of ambient temperature slows down many biosynthetic processes, including synthesis of FA, forcing modification of already existing fatty acids in membrane lipids to adapt to changes in fluidity. Such modification includes desaturation by three fatty acid desaturation enzymes, which add double bonds to FA thus increasing fluidity previously lowered by cold [43]. Evidently, the transcription of genes encoding these FADs (*desA* for Δ12-desaturase, *desB* for ω3-desaturase, and *desD* for Δ6-desaturase) is induced by cold stress [44]. In addition, RbpA3 secures the stability of mRNA of these genes [45].

(5) cofactors and nucleotide metabolism, other metabolic functions;

(6) genes with unspecified functions.

It should be noted that some cold-induced genes strictly depend on light, and are never induced in the dark: *hliA* and *hliB* [46,47] *ycf39* for a PS II assembly factor [48], *lilA* [49], and several genes encoding proteins involved in redox-sensitive regulation: *ndhD2*, *pedR* and *gshB* [50,51,52].

While more than 100 genes induce their transcription in response to cold, only 30 of them are cold-specific and do not respond to any other stresses. Together with genes of unspecified functions, this group includes genes that encode proteins involved in transcription and translation (*rbpA*-family, *smpB*, *rplT*, *pfbB*), methylation (*mbpA*), and metabolism (*cbiF*, *ams1*).

The other two thirds of cold-induced genes react to additional two or more abiotic stresses (Table 2).

The induction of the expression of the same genes by different stressors suggests the existence of some common, universal stress components. In photosynthetic cells or organisms, such components are hypothesized to be related to changes in oxidative state of cellular components. The mechanism of their action would include either electric or chemical signaling, specifically by reactive oxygen species [53].

### 2.5. Redox Regulation and Oxidative Stress

The redox status of a cell is important for DNA synthesis, gene expression, and enzymatic activity. The redox homeostasis is mainly maintained by thiol-containing molecules such as glutathione, thioredoxins, glutaredoxins, and peroxiredoxins. Stress-induced changes in the redox status of thylakoid membranes directly affect the function of the photosynthetic electron transport chain (ETC).

The majority of the GSP genes are regulated, among other stresses, by the redox status of the plastoquinone (PQ) pool. The status changes as electrons are transferred through the ETC. Such changes are experimentally achieved with the chemicals that interrupt electron flow either upstream (DCMU) or downstream (DBMIB) of the PQ pool. Alternatively, strong light [54] or acidification [27] may alter the Q_B_ redox status that affects gene transcription. For example, the reduction of PQ by DBMIB causes the induction of the GSP genes [12].

Expression of GSP genes is also induced by reactive oxygen species (ROS), which are formed during stress and change the oxidative state of a cell. Of all various forms of ROS, summarized in [52], hydrogen peroxide (H_2_O_2_) is considered the most important in oxidative stress due to its reasonable lifetime allowing it to perform regulatory functions.

H_2_O_2_ in characterized by relatively long lifetime, and is moderately reactive. However, it might get reduced to form hydroxyl radical via a Fenton reaction and, thus, become destructive for cells. Oxidative stress (0.25 mM H_2_O_2_) in a liquid culture of *Synechocystis* induces transcription of ~100 genes with IF ≥ 3 [14], among which there are GSP genes. Similarly to heat and other stresses, ROS also induce genes that are necessary for stress neutralization: genes encoding thioredoxin (*trx*), peroxiredoxin (*aphC*), and catalase (*katG*). H_2_O_2_ specifically induces transcription of *perR* (*slr1738*) for the transcriptional regulator PerR [38] that is directly regulated by hydrogen peroxide via oxidation of His^37^ and His^91^ residues, which leads to dissociation of PerR-DNA complex [55].

Overall analysis of cyanobacterial stress transcriptome suggests that H_2_O_2_ and redox changes in ETC (specifically in PQ redox state), commonly present in most abiotic stresses, are the universal triggers of stress responses in photosynthetic bacteria. Further, we discuss experimental evidence to support this hypothesis.

## 3. The Redox State of Plastoquinone (PQ) Depends on Membrane Fluidity

Temperature fluctuations cause significant changes in membrane fluidity, which in turn (1) activates membrane receptors or (2) affects membrane-related metabolic processes.

Transmembrane receptors that perceive cold stress in bacteria are histidine kinases (Hiks). Hiks sense a change in thickness of the cytoplasmic membrane due to changes in its viscosity [56]. In cyanobacteria, about a half of the cold-sensitive genes are controlled by the red-light-dependent transmembrane histidine kinase Hik33 [47,57], which also partially controls the reactions to hyperosmotic, salt, and oxidative stress [1]. In general, the cold-altered membrane fluidity can be restored via FA desaturation by FADs, a process similar in all eubacteria and plants.

Photosynthesis and respiration are two major metabolic processes located in membranes thus affected by its physical state. Structural and functional organization of photosynthetic apparatus in cyanobacteria is similar to that in higher plants. In cyanobacteria, however, water is not the only electron donor. Some substrates are oxidized by respiratory ETC enzymes, which interact with the photosynthetic ETC via PQ. The redox state of PQ determines the activity of photosynthetic ETC and redistribution of energy between PS II and PS I. Since interaction of PQ with ETC components is a diffusion-driven process, the fluidity of thylakoid membranes is a key factor defining the efficiency of these interactions. In turn, the membrane fluidity depends on the proportion of saturated and (poly)unsaturated FAs that are produced by FADs [43]. X-ray crystallography of cyanobacterial PS II and PS I clearly indicate the presence of a significant amount of lipids in photosynthetic reaction centers that may participate in structuring and regulation of electron transfer [58].

*Synechocystis* cell culture grown at its optimal temperature (30–35 °C), is able to adapt to cold (20–22 °C) by adjusting its membrane fluidity via induction of several FADs which in turn increase the amount of unsaturated fatty acids in membrane lipids.

**Figure 2:**
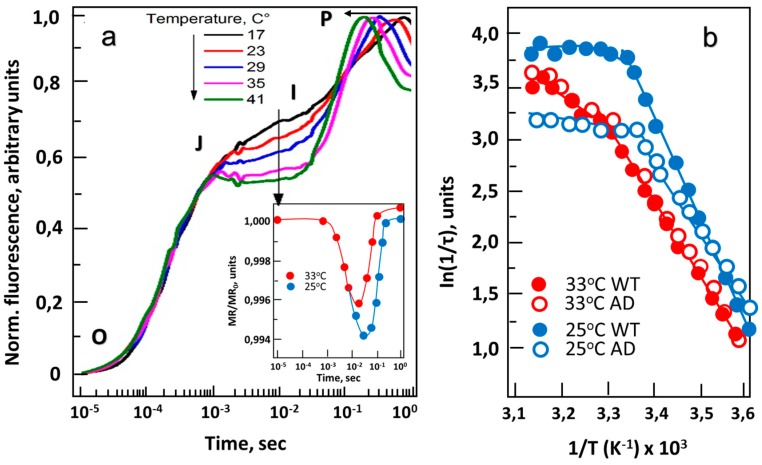
The effect of temperature on chlorophyll fluorescence induction in *Synechocystis*. (**a**) Wild-type (WT) strain. Characteristic induction curves obtained at 17, 23, 29, 35 and 41 °C; letters O, J, I and P indicate different stages of plastoquinone (PQ) reduction, according to standard nomenclature. Insert panel: the effect of temperature on kinetics of modulated reflection (MR) at 820 nm obtained at 25 and 32 °C. (**b**) Arrhenius plots of P700^+^ reduction rates of wild-type strain (WT, solid symbols) and *desA*^−^/*desD*^−^ mutant (AD; open symbols) at 33 °C (red circles) and 25 °C (blue circles). Cell cultures were dark-adapted for 5 min prior to MR measurements. MR changes were induced by array of red (627 ± 10 nm) light-emitting diodes (LEDs) delivering 3000 µmol photons/m^2^ sec of actinic light to a sample [59].

Assuming that differences in membrane fluidity affect the diffusion rate of PQ and, accordingly, the electron transfer rate at the donor side of PS I, one would expect both to change under cold stress. Evidence suggests this hypothesis to be true [59]. In the experiment where redox activity of photosystems was measured at different temperatures using modulated reflection (MR), it was observed that initially (100 µs–10 ms), the reduction rate of P700^+^ does not depend on temperature in a range of 5–45 °C, but strongly depends on the density of photon flux (Figure 2a), indicating that MR is controlled by photo-activated formation of P700^+^. However, later (10^−2^–10 s) the MR signal strongly depends on temperature, but not on the photon flux density (Figure 2a). This means that the reduction of P700^+^ depends on the rate of oxidation of PQ by cytochrome *b*_6_*f* complex, which is a diffusion-driven process. In addition, during cold treatment at 25 °C, the reduction of P700^+^ occurs much faster in wild-type (WT) than in mutant cells defective in genes for cold-induced FADs (Figure 2b), since the mutant is not able to compensate for membrane rigidification caused by a decrease in temperature [59].

At optimal temperature of 32 °C, wild type and double-mutant (*desA*¯/*desD*¯) *Synechocystis* with inactivated Δ12- and Δ6-FADs (hereafter – AD cell culture) show similar reduction rates of P700^+^. However, at 25 °C, AD cell culture had significantly lower reduction rates of P700^+^ compared to WT cells (Figure 2b), demonstrating the importance of unsaturated FAs for regulating ETC under cold stress [57]. It turns out that at low temperatures, regulation of the redox state of the PQ pool strongly depends on the fluidity of the membrane controlled by cold-induced Δ12- and Δ6-FADs.

The correlation between FAs unsaturation and membrane fluidity have been established by different approaches including differential scanning calorimetry [60], Fourier transform [15] and Raman [59] spectroscopy, measurements of steady-state fluorescence anisotropy with 1,6-diphenyl-1,3,5-hexatriene (DPH) [46] and other lipid-phase intercalating probes. All experimental evidence suggests significant difference in fluidity of thylakoid and cytoplasmic membranes between wild-type and AD cell cultures. Thus, the oxidation rate and diffusion coefficient of PQ, as well as the exchange rate of electrons between the PQ and PS II, all depend on membrane fluidity, which is mainly regulated by the FADs.

**Figure 3:**
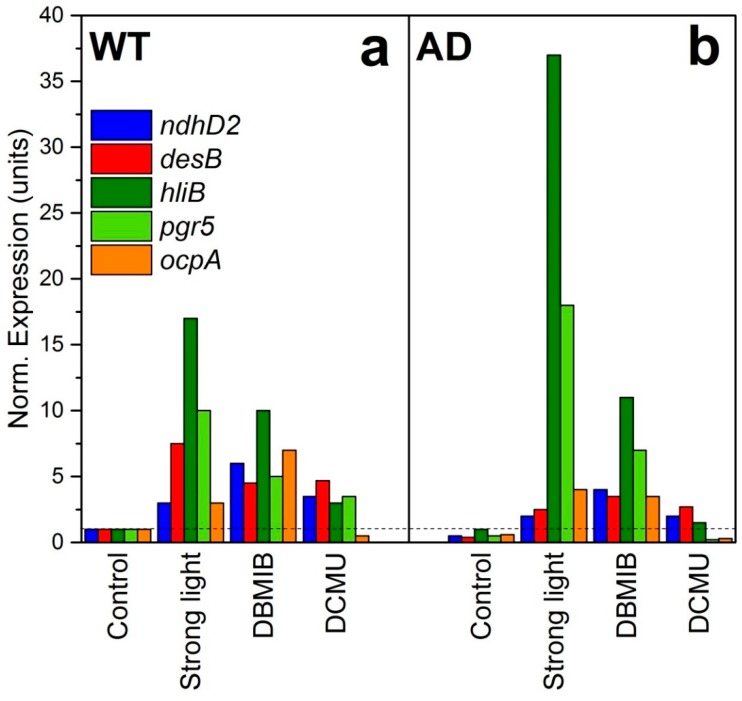
Gene expression in WT (**A**) and *desA*^−^/*desD*^−^ (=AD) (**B**) cells grown at 32°C under illumination of 70 µmol photons/m^2^ sec and exposed for 30 min to strong high light (2500 µmol photons/m^2^ sec), or treated with 10 µM 2,5-dibromo-3-methyl-6-isopropyl-*p*-benzoquinone (DBMIB) or 10 µM 3-(3,4-dichlorophenyl)-1,1-dimethylurea (DCMU). Reproduced from [59] with permission.

Consequently, fluidity-dependent changes in redox potential of PQ should trigger cellular responses to cold stress. To confirm this hypothesis, redox changes in PQ pool have been stimulated at optimal growth temperature by the ETC inhibitors, such as 3-(3,4-dichlorophenyl)-1,1-dimethylurea (DCMU or diuron, an oxidizer of PQ) and dibromotiminoquinone (DBMIB—the reducer of PQ). Diuron blocks the transfer of electrons from PS II, which leads to the oxidation of the following ETC components, including PQ. DBMIB acts in the region of the Qo site of cytochrome *b*_6_*f* and inhibits electron transfer to Cyt *b*_6_*f*, thus, reducing PQ. The experimental treatments with DCMU and DBMIB were conducted in WT and AD cells (with rigidified membranes) grown at optimal temperature (32 °C). Simultaneously, expression of several cold-responsive genes, including *ndhD2* (NADH-dehydrogenase) and *desB* (terminal ω3-FAD) has been measured [59]. The expression of these two genes is controlled by Hik33 and also depends on both light and membrane fluidity. It was found, that at optimal temperature of 33 °C, the *ndhD2* gene is induced by strong light and DCMU in both WT and AD cell cultures, but induction in AD is markedly lower. Treatment with DBMIB induced expression of *ndhD2* and *desB* to rather a similar extent in both WT and AD cells. The results suggest that redox-dependent transcription of these two genes is less sensitive to PQ oxidation by strong light and DCMU in cells with rigidified membranes, whereas DBMIB-induced PQ reduction does not produce a significant difference between these two strains (Figure 3).

Taking into consideration that cold-treated WT cells are able to compensate for the PS II dependent reduction of PQ and maintain the H^+^ gradient via the respiratory ETC, while AD mutant cells are not, one may expect the relevant differences in cellular stress responses.

Besides *ndhD2* and *desB*, expression of other stress-responsive genes was affected by PQ redox state. *HliB* and *pgr5* genes were highly induced in AD cells, and their transcription was regulated by PQ reduction (Figure 3). Both are shown to be induced by strong light and cold shock; HliB is associated with PSI trimers and protects it under stress conditions [46], and *pgr5* encodes an analog of a higher plants proton gradient regulation 5 protein (Prg5) with a controversial function [61]. Expression of another gene, *ocpA*, was induced by DBMIB, but to lower extent in AD cells to compare to WT cells. Orange carotenoid protein (OCP) participates in non-photochemical quenching in cyanobacterial cells. Overall, the evidence suggests that PQ reduction affects the transcription of genes related to stress regulation (Figure 3).

Apparently, oxidation and reduction of plastoquinone caused by changes in temperature-dependent fluidity of membranes affect transcription of a vast number of stress genes. The redox state of plastoquinone must work as a key signal turning on the expression of FADs and consequent desaturation of fatty acids in membranes. Change of PQ oxidative state is a good candidate for a role of a common component connecting light, cold or other stresses.

An alternative experiment, where instead of membrane rigidification via targeted FAD mutations, various alcohols were applied to cells as membrane “fluidizers”, was also performed earlier [62]. Expression of GSP and FAD genes expression was measured. Hexanol (hexan-1-ol, C6) was found to have the strongest fluidizing effect on membranes among all alcohols (C1-C9) studied. It was found that the effectiveness of hexanol correlates with both duration of exposure and concentration of alcohol in the environment. The spectrum of hexanol-inducible genes generally coincides with the spectrum of genes induced by aromatic benzyl alcohol or heat stress [62]. This suggests again [63,64] that a change in the membrane fluidity may serve as a primary signal for triggering stress responses, probably through a change in the redox potential of the quinone pool. This, in turn, may lead to emergence of long-lived ROS in the form of hydrogen peroxide, which can serve as a universal chemical trigger for launching stress-protective systems.

## 4. H_2_O_2_ is Involved in Regulation of Cold Stress Responses

To test the hypothesis on H_2_O_2_ as a universal trigger of stress responses, a double-mutant of *Synechocystis*, simultaneously defective in two antioxidant enzymes, catalase-peroxidase (KatG) and thioredoxin-peroxidase (Tpx) [65] was used to study transcription under oxidative stress [66]. The mutant was necessary to perform such analysis since due to a rapid detoxification of peroxide under normal conditions, its effect is not traceable in the wild-type cyanobacterium [66].

**Figure 4:**
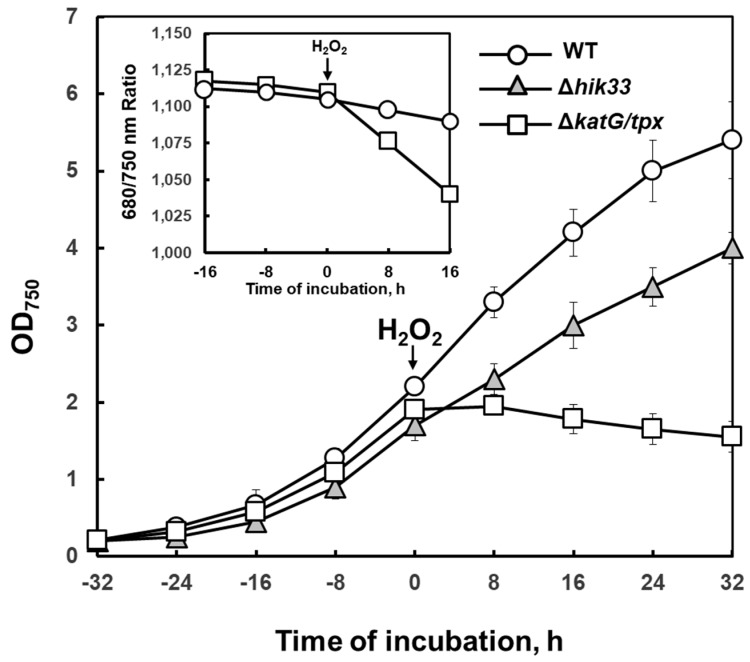
The effect of exogenous hydrogen peroxide on *Synechocystis* growth at 32 °C. Point 0: H_2_O_2_ was added to a final concentration of 0.25 mM. Optical density measured at 750 nm reflects cell growth. Insert panel: ratio of absorbance at 680/750 nm, characteristic of the relative content of chlorophyll in cells. Reproduced from [66] with permission.

It was shown that such mutant has a delayed detoxification mechanism, which gives plenty of time to detect changes in gene expression. Unlike WT, mutant cells cannot grow in the presence of exogenous H_2_O_2_. The *katG*/*tpx* mutant is extremely sensitive to low concentrations of H_2_O_2_, especially under low temperature stress (Figure 4).

**Figure 5:**
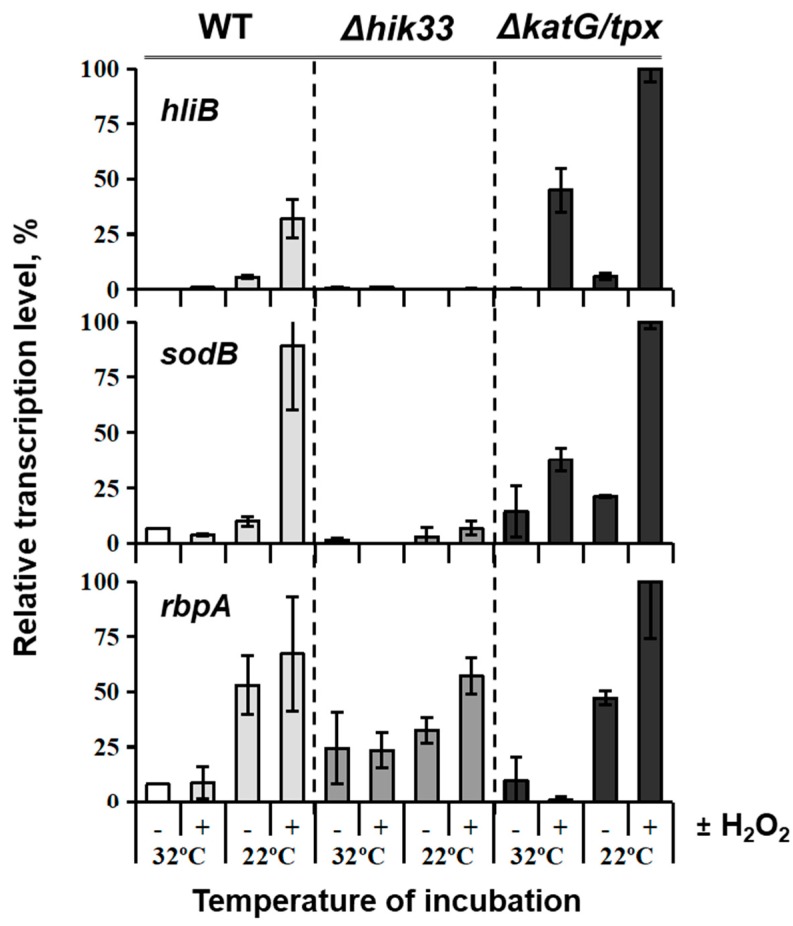
Analysis of stress-inducible gene transcription in *Synechocystis* strains. Wild-type (WT), Δ*hik33* mutant, and Δ*katG/tpx* double mutant strains were grown at 32 °C (control), then exposed to 22 °C for 30 min in the presence (+) or absence (−) of H_2_O_2_ at a final concentration of 0.25 mM. Control samples (32 °C) were also subjected to 0.25 mM H_2_O_2_. Transcription of Hik33-dependent (*hliB*, and *sodB*) and Hik33-independent (*rbpA*) genes was analyzed. Amounts of transcripts were assessed by qRT-PCR, normalized to those of the housekeeping gene *rnpB*, and expressed in % of the maximum value of gene expression in all samples [66].

Subjected to both treatments with H_2_O_2_ and low temperatures, use of the double-mutant managed to demonstrate that peroxide is involved in regulation of genes induced by cold (Figure 5) in a Hik33-dependent manner [66]. This connection was made based on the fact that cold-inducible Hik33-regulated genes, *hliB* and *sodB*, were stimulated by H_2_O_2_ in a wild type strain, while in the Δ*hik33* mutant these genes were not induced by cold anymore despite the presence or absence of hydrogen peroxide. Hik33-independent *rbpA*, however, served as control in this experiment and responded to cold stress uniformly in both WT and mutant [66]. Apparently, Hik33 is sensitive to ROS such as H_2_O_2_, which explains its general involvement in the sensing of stresses where reactive oxygen species are present, with light being the major stimulant of ROS formation.

An additional conformation of this statement recently came from experimental studies on RpaB, a cognate response regulator for Hik33, which is shown to interact with ~150 target light-regulated promoters [67]. RpaB is suggested to pair with Hik33 to sense ROS, controlling expression of genes involved in diverse photosynthetic functions, e.g., photoprotection, cyclic electron flow and state transitions, photorespiration, etc. Similarly to Hik33, RpaB controls responses to strong light, cold stress, and iron deficiency [7,66,67].

In *Synechocystis*, an interaction between H_2_O_2_ and either regulatory Hiks [14], transcription factors [50,51,67], or σ-factors of RNA polymerase [68] has been demonstrated. Two important participants in oxidative stress response have been identified as non-essential group 2 σ-factors, SigB and SigD (see Section 2 of this article). Both of them belong to GSPs. Cells that lack SigB and SigD fail to acclimate to high light, singlet oxygen and H_2_O_2_, while the overproduction of these σ-factors leads to superior growth of cells under those stress conditions [68].

The question of the exact mechanism of H_2_O_2_ action is still being discussed. Presumably, it acts on proteins as an oxidant, affecting their folding and activity [69,70,71], starting multiple protective pathways. Interestingly, Hik33 of *Synechocystis* does not contain amino acid residues that might be oxidized by H_2_O_2_, rather it contains specific light-dependent electron-transport domains PAS and HAMP that might participate in redox-regulated signal transduction [72,73], that are likely to interact with PQ which in turn is sensitive to H_2_O_2_.

## 5. Conclusions

Despite the significant amount of research on cyanobacterial stress response, certain aspects of the latter have remained unclear until the present day. A considerable amount of data on stress-induced transcriptome in *Synechocystis* suggest that there is no simple relationship between one stress type, one stress sensor and one specific gene or a group of genes changing expression in response. The fact that one sensor can perceive signals from multiple stresses, and that expression of certain groups of genes is not specific to one type of stress, has been puzzling researchers for a long period of time. In certain stresses, such as cold, hyperosmotic and salt stress, a common component whose action results in a comparable cellular response can be identified relatively easily as a decrease in cell volume and rigidification of the membrane. Here, we suggest reactive oxygen species, and specifically H_2_O_2_, to be another common component interconnecting several types of abiotic stresses. In summary, we conclude that: (1) H_2_O_2_ and redox status of PQ are the universal components of various stresses that trigger similar responses in cyanobacterial cells; (2) ROS formation (in particular, H_2_O_2_) depends on the physical properties of both cytoplasmic and thylakoid membranes; (3) the destructive effect of H_2_O_2_ is reduced by increasing of fluidity of biological membranes.

## Figures and Tables

**Table 1:** Stress-induced genes of *Synechocystis* sp. PCC 6803: heat stress and other stressors.

				Abiotic Stressors						Redox		ROS
ORF	Gene	Protein	Function	Heat	Salt	Osmo	Light	UV-B	pH	DBMIB	DCMU	H_2_O_2_
***slr1285***	*hik34*	Hik34	Sensor histidine kinase Hik34	+	+	+		+	+	+		+
***sll0306***	*sigB*	SigB	Sigma factor B of RNA polymerase	+	+	+		+	+	+		+
***slr2075***	*groES*	GroES	Heat-shock protein (HSP) 10 kDa co-chaperonin	+	+	+	+	+		+		
***slr2076***	*groEL1*	GroEL1	HSP 60 kDa chaperonin 1	+	+	+	+	+		+		
***sll0416***	*groEL2*	GroEL2	HSP 60 kDa chaperonin 2	+	+	+	+					
***sll1514***	*hspA*	HspA	HSP 17 kDa	+	+	+	+	+	+	+		+
***sll0170***	*dnaK2*	DnaK2	HSP 70 kDa	+	+	+	+	+	+	+		+
***slr0093***	*dnaJ*	DnaJ	HSP 40 kDa	+	+	+		+	+	+		+
***sll0430***	*htpG*	HtpG	HSP 90 kDa	+	+	+	+	+		+		
***slr1641***	*clpB1*	ClpB1	HSP 100 kDa chaperone	+	+	+	+	+		+		+
***slr1204***	*htrA*	HtrA	Serine protease	+	+	+		+	+	+		+
***slr0008***	*ctpA*	CtpA	C-terminal processing protease	+			+	+	+			+
***sll1621***	*sll1621*	PrxA	Peroxiredoxin	+	+		+	+		+		+
***slr0095***	*smtA*	SmtA	*S*-adenosyl methionine methyltransferase	+	+		+			+		+
***slr1674***	*slr1674*	Slr1674	Thermoprotector protein of PS II	+	+		+	+	+	+		+
***slr1963***	*ocpA*	OCP	Orange carotenoid-binding protein	+		+	+	+		+		
***slr1512***	*sbtA*	SbtA	Na-dependent bicarnonate transporter	+			+					+
***slr1516***	*sodB*	SodB	Superoxide dismutase	+	+	+	+	+	+			
***sll0528***	*sll0528*	Sll0528	Site-2-protease	+	+	+	+	+	+	+		+
***slr1675***	*hypA1*	HypA1	Hydrogenase maturation factor HypA1	+	+	+	+	+	+	+		+
***ssl3044***	*ssl3044*	Fdx	Ferredoxin	+	+	+	+	+	+	+		+
***slr1687***	*nblB1*	NblB1	Phycobilisome degradation protein	+	+	+	+	+	+	+	+	+
***sll0939***	*sll0939*	Sll0939	Low pH resistance protein	+	+	+		+	+	+	+	+
***slr0967***	*slr0967*	Slr0967	Low pH resistance protein	+	+	+		+	+	+	+	+
***slr1686***	*slr1686*		Unknown function	+	+		+	+				+
***slr1603***	*slr1603*		Unknown function	+	+	+		+		+		+
***sll1853***	*sll1853*		Unknown function	+	+					+		
***sll0846***	*sll0846*		Unknown function	+	+	+	+	+	+	+		+
***sll1884***	*sll1884*		Unknown function	+	+	+	+					+
sll1009*	frpC*	FrpC	Membrane Fe-regulated protein	+								
sll1501*	cbiA*	CbiA	Cobalamine biosynthesis protein	+								
sll0441*	sll0441*		Unknown function	+								
sll1892*	sll1892*		Unknown function	+								
slr0670*	slr0670*		Unknown function	+								
sll0982*	sll0982*		Unknown function	+								
slr1127*	slr1127*		Unknown function	+								

* Genes induced only by heat stress. ORF—open reading frame. Heat—heat stress (44 °C for 15–20 min); Salt—salt stress (0.5 M NaCl for 15–20 min); Osmo—hyperosmotic stress (0.5 M sorbitol for 15–20 min); Light—change of light intensity (from 20 to 300 µmol quanta/m^2^ sec for 30 min); UV-B—ultraviolet (UV-B) light for 30 min; pH—low pH ~4.0 (30 min); DBMIB—10 µM 2,5-dibromo-6-isopropyl-3-methyl-1,4-benzoquinone (30 min); DCMU—10 µM 3-(3,4-dichlorphenyl)-1,1-dimethylurea (30 min); H_2_O_2_—250 µM hydrogen peroxide (20 min).

**Table 2:** Stress-induced genes of *Synechocystis* sp. PCC 6803: cold stress and other stressors.

ORF	Gene	Protein	Function	Abiotic stressor						Redox		ROS
				Cold	Salt	Osmo	Light	UV-B	pH	DBMIB	DCMU	H_2_O_2_
***sll0790***	*hik31*	Hik31	Two-component sensor histidine kinase	+						+		
***slr1594***	*rre5*	Rre5	Response regulator	+			+			+		
***slr0083***	*crhR*	CrhR	RNA helicase	+	+	+	+			+		
***sll0517***	*rbpA1*	RbpA1	RNA binding protein A1	+								+
***sll0533***	*tig*	Tig	Ribosome trigger factor	+		+	+					
***sll1096***	*rpsL*	Rps12	30S ribosomal protein S12	+		+	+					
***slr0082***	*rimO*	RimO	Methyltransferase of ribosomal protein S12	+	+	+	+			+		
***slr1105***	*typA*	TypA	GTP-binding protein TypA/BipA	+		+	+			+		+
***slr1512***	*sbtA*	SbtA^1^	Na-dependent bicarbonate transporter	+			+					+
***sll1541***	*syc2*	Syc2	Carotenoid oxygenase	+	+		+	+		+		+
***slr1291***	*ndhD2*	NdhD2	NADH dehydrogenase subunit 4	+			+	+		+		+
***ssl2542***	*hliA*	HliA	High light inducible protein	+	+	+	+	+		+		+
***ssr2595***	*hliB*	HliB	High light inducible protein	+	+	+	+	+	+	+	+	
***slr1544***	*lilA*	LilA	Light-harvesting protein LilA	+	+	+	+	+	+	+	+	+
***slr1687***	*nblB*	NblB	Phycocyanobilin lyase NblB	+	+	+	+	+	+	+	+	+
***sll1742***	*nusG*	NusG	Transcription antitermination protein	+	+	+	+					+
***sll1818***	*rpoA*	RpoA	RNA polymerase alpha subunit σ^70^	+		+	+					
***sll2012*^1^**	*sigD* ^1^	SigD^1^	RNA polymerase sigma factor SigD	+	+		+	+	+	+	+	+
***ssl3044*^1^**	*ssl3044* ^1^	Fdx^1^	Ferredoxin	+	+	+	+	+	+	+		+
***slr1350***	*desA*	DesA	Δ12 fatty acid desaturase	+			+					
***sll1441***	*desB*	DesB	ω3 fatty acid desaturase	+			+					
***slr1992***	*gpx2*	Gpx2	Hydroperoxy fatty acid reductase	+			+	+				
***sll1483***	*sll1483*		Salt-induced periplasmic protein	+	+	+	+	+	+	+	+	+
***sll0157***	*sll0157*		Zn-dependent hydrolase	+	+	+		+		+		
***sll1863***	*sll1863*		Unknown protein	+	+	+						+
***sll1862***	*sll1862*		Unknown protein	+	+	+						+
***sll1853***	*sll1853*		Unknown protein	+	+					+		
***slr0551***	*slr0551*		Unknown protein	+		+	+	+		+	+	
***slr0959***	*slr0959*		Unknown protein	+	+		+	+				
***slr1686***	*slr1686*		Unknown protein	+	+		+	+				+
***slr1687***	*slr1687*		Unknown protein	+	+		+	+				+

ORF—open reading frame; Cold—cold stress (22°C for 30 min); Salt—salt stress (0.5 M NaCl for 15–20 min); Osmo—hyperosmotic stress (0.5 M sorbitol for 15–20 min); Light—strong light (a shift from 20 to 300 μmol photons/m^2^ s); UV—exposure to UV-B for 30 min; pH—lower pH from 8.0 to 4.0 for 30 min; DBMIB—10 μM 2,5-dibromo-6-isopropyl-3-methyl-1,4-benzoquinone; DCMU—10 μM 3-(3,4-dichlorophenyl)-1,1-dimethylure; H_2_O_2_—0.25 mM hydrogen peroxide. Reproduced from [5] with permission.
